# Elevated serum cystatin C predicts tophus formation in gout: evidence from a cross-sectional study

**DOI:** 10.3389/fendo.2026.1733216

**Published:** 2026-03-30

**Authors:** Yonglu Hu, Chengqian Li, Tian Liu, Jie Lu, Aichang Ji, Yangang Wang, Bingzi Dong

**Affiliations:** 1 Department of Endocrinology and Metabolism, The Affiliated Hospital of Qingdao University, Qingdao, China; 2 Shandong Provincial Clinical Research Center for Immune Diseases, The Affiliated Hospital of Qingdao University, Qingdao, China

**Keywords:** cystatin C, tophus, bone erosion, magnesium, gout

## Abstract

**Objective:**

To investigate the association between serum cystatin C (CysC) levels and tophus presence, and to assess its diagnostic value for structural joint damage in gout.

**Methods:**

In this cross-sectional study of 598 gout patients, we examined the association between serum CysC and both the presence and burden of tophi. The predictive value of CysC for tophus and bone erosion was further evaluated using logistic regression and ROC curve analyses.

**Results:**

Elevated serum CysC levels were significantly associated with older age, longer disease duration, and an increased prevalence of tophus, bone erosion, and the patients in the highest quartile (Q4, >1.28 mg/L) had a 3.87-fold higher adjusted odds of tophus (P < 0.05), showing a significant dose–response trend (P for trend = 0.003). Serum CysC alone demonstrated a moderate predictive value for tophus (AUC = 0.627), which improved in multivariable models (AUC = 0.777). A similar enhancement in predictive performance was noted for bone erosion (fully adjusted AUC, 0.756). There was a significant interaction between serum CysC and magnesium regarding tophus risk (P < 0.001). Furthermore, multivariate ordinal regression analysis identified higher CysC, longer gout duration, bone erosion, and the double contour sign as independent predictors of tophus burden.

**Conclusion:**

Serum CysC independently predicts tophus in gout and shows interaction with serum magnesium. Its integration into risk assessment may improve clinical stratification and support timely, individualized treatment strategies.

## 1 Introduction

Gout is a metabolic disorder caused by impaired purine metabolism, affecting approximately 56 million individuals worldwide as of 2020. With ongoing population growth and socioeconomic development, its global prevalence is projected to reach 96 million by 2050 (1, 2). As the most common metabolic arthritis, gout is characterized by the accumulation of monosodium urate (MSU) crystals in joints, resulting in acute arthritis, chronic structural damage, and tophus formation (3). Recurrent flares, particularly in the presence of tophi, can significantly compromise joint function and reduce patients’ quality of life. In severe cases, patients may even require surgical intervention (4). The condition predominantly affects men, with prevalence rising progressively with advancing age (2). Gout is widely recognized as a key contributor to renal impairment. In patients with coexisting chronic kidney disease (CKD), crystal deposition is commonly observed in renal tissue. Histological studies suggest that such deposits, often surrounded by inflammatory infiltrates and associated with interstitial fibrosis, may contribute to tubulointerstitial nephritis and progressive renal dysfunction (5, 6). According to a meta-analysis in the UK, 24% (95% CI, 19–28) of patients with gout exhibited an eGFR < 60 mL/min/1.73 m² (7). Reduced eGFR also contributes to the accumulation of serum uric acid (SUA), thereby increasing the risk of gout flares and creating a vicious cycle of disease progression (8). Elevated SUA activates NADPH oxidase, inducing oxidative stress and promoting the generation of reactive oxygen species. In parallel, SUA upregulates proinflammatory mediators, triggering systemic inflammation that impairs endothelial function, promotes vascular remodeling, and fosters insulin resistance, collectively driving the development and progression of cardiorenal-metabolic disease (9–11).

Cystatin C (CysC), a sensitive endogenous biomarker of kidney filtration function, demonstrates superior accuracy in eGFR compared to serum creatinine, particularly during early renal impairment (12). Furthermore, CysC levels exhibit minimal confounding influence from age, sex, or muscle mass, unlike creatinine (13). This enhanced reliability stems from its constitutive production by nucleated cells and exclusive glomerular filtration without tubular secretion, making elevated serum CysC a direct indicator of even minimal GFR reductions (14). In patients with gout, MSU crystal deposition in renal tissue may contribute to tubulointerstitial injury and progressive renal impairment. Despite its clinical potential, few studies have examined the relationship between serum CysC levels and the clinical progression of gout, particularly concerning tophus formation.

In this study, we investigated the association between serum CysC levels and tophus formation in male patients with gout, aiming to provide new insights into its clinical utility as a marker of both renal function and disease burden.

## 2 Materials and study design

### 2.1 Study population

A total of 598 male patients with gout were enrolled at the Affiliated Hospital of Qingdao University. Between August 2023 and January 2025, patients diagnosed with gout were consecutively recruited based on the 2015 ACR/EULAR gout classification criteria. The inclusion criteria were as follows (1): male sex; (2) age between 18 and 80 years; (3) eGFR ≥60 mL/min/1.73 m²; (4) history of at least one documented gout flare or currently experiencing an acute gout attack; and (5) all patients underwent dual-energy CT (DECT) scans and ultrasonography of joints including elbows, wrists, interphalangeal joints, knees and ankles. Exclusion criteria included: (1) age <18 or >80 years; (2) female sex; (3) eGFR <60 mL/min/1.73 m²; (4) occurrence of urinary tract infection or other inflammatory diseases, whether acute or chronic; (5) any liver abnormalities, autoimmune disease, hematologic disorders, malignancies, or psychiatric conditions; (6) use of medications that may affect uric acid metabolism or renal stone formation—such as potassium citrate, allopurinol, or thiazide diuretics—within the past three months; and (7) presence of other inflammatory arthritides, such as rheumatoid arthritis, ankylosing spondylitis, or psoriatic arthritis. The Ethics Committee of the Affiliated Hospital of Qingdao University approved the study (QYFY WZLL 30274). Written informed consent was obtained from all participants.

### 2.2 Data collection

We collected clinical parameters including age, sex, body mass index (BMI), blood pressure, duration of gout, and current smoking. BMI was determined as the ratio of weight (kg) to height squared (m²). After an overnight fast, venous blood samples were collected in the early morning to measure serum levels of CysC, fasting plasma glucose (FPG), total cholesterol (TC), triglycerides (TG), low-density lipoprotein cholesterol (LDL-c), high-density lipoprotein cholesterol (HDL-c), free fatty acids (FFA), serum creatinine (Scr), serum uric acid (SUA), serum magnesium (Mg), and other routine biochemical indicators. Quantitative analysis of CysC was performed using a latex-enhanced immunoturbidimetric assay with reagents provided by Fosun Diagnostics on a Beckman Coulter AU5800 analyzer. The eGFR was calculated based on the Chronic Kidney Disease Epidemiology Collaboration (CKD-EPI) formula (15).

### 2.3 Assessment of tophi

All patients underwent comprehensive DECT examinations using a second-generation dual-source CT scanner (Somatom Definition Flash, Siemens Healthineers) targeting multiple joints, including bilateral wrists, shoulders, ankles, interphalangeal joints, first metatarsophalangeal joints, and knees. Image datasets were obtained using a Syngo Multimodality Workplace workstation (Siemens Healthineers) and reconstructed with dedicated gout analysis software (DE Gout, Siemens Healthineers). Scans were categorized as positive or negative based on the presence of MSU crystals, which were pseudocolored green in post-processed images. All studies were independently evaluated by a musculoskeletal radiologist with 5 years of DECT experience, blinded to clinical and ultrasonographic data. MSU deposition sites were recorded following standardized partitioning criteria on cross-sectional reconstructions, and deposition volumes were quantified automatically after manual delineation of the image range. Common artifacts—including nailbed, submillimeter, cutaneous, motion, beam-hardening, and vascular artifacts—were systematically excluded in accordance with ACR/EULAR guidelines. Tophi were identified based on the 2012 ACR criteria (16, 17). Indeterminate cases were independently reviewed by a second radiologist to reach a diagnostic consensus.

### 2.4 Assessment of bone erosion

Bone erosions on DECT, defined as cortical disruption with contour discontinuity in ≥2 orthogonal planes, were independently evaluated by two blinded musculoskeletal radiologists with 5 years of DECT experience, without reference to laboratory data (18).

### 2.5 Statistical analysis

Statistical analysis was conducted using SPSS version 27.0 and R version 4.4.2. Continuous variables were expressed as mean ± standard deviation (SD) or median with interquartile range (IQR), depending on data distribution. Normality was assessed before analysis. Categorical variables were presented as percentages. Participants were categorized into quartiles based on serum CysC levels: Q1 (≤0.93 mmol/L), Q2 (0.93–1.06 mmol/L), Q3 (1.06–1.28 mmol/L), and Q4 (>1.28 mmol/L), with Q1 serving as the reference group.

Categorical variables across quartiles were compared using χ^2^ tests. Inter-group comparisons of continuous variables employed Student’s t-tests or Mann-Whitney U tests. Multi-group comparisons utilized one-way ANOVA or Kruskal-Wallis H tests. Generalized linear models (GLM) with an inverse Gaussian distribution and a log link were applied to examine the associations between CysC and clinical characteristics. Logistic regression analysis was used to evaluate factors associated with tophus formation. A two-tailed P-value < 0.05 was considered statistically significant. Model stability and internal validity were assessed using 100 bootstrap samples, and calibration curves evaluated agreement between predicted and observed probabilities.

Using R (version 4.4.2), we developed a simplified risk score for tophus formation based on CysC, serum Mg, gout duration, and the CysC–Mg interaction, with regression coefficients scaled into an integer-based point system.

## 3 Results

### 3.1 Clinical characteristics of enrolled patients with or without tophi

The study included 598 male gout patients, of whom 459 had tophi and 139 did not (**Table 1**). Compared with those without tophi, patients with tophi were significantly older and had a longer gout duration. They were more likely to be current smokers and had a higher prevalence of kidney stones, bone erosion, double contour sign, and joint effusion. Additionally, the number of joints affected by gout was significantly greater in the tophi group (all P<0.001). Levels of TC, TG, LDL-C, and FPG were substantially higher, whereas serum creatinine–based eGFR was lower in patients with tophi (P = 0.002). Notably, serum CysC was strongly associated with the presence of tophi (P<0.001).

**Table 1:** Clinical characteristics of gout patients with and without tophi.

Variables	Tophus (n=459)	Tophus-free (n=139)	t/Z/χ^2^ value	P- value
Age (years)	52.00 (40.00, 62.00)	48.00(34.00, 62.00)	-2.136	0.033*
Duration of gout (years)	8.00 (4.00, 14.00)	3.00 (0.33, 7.00)	-7.199	<0.001*
BMI (kg/m^2^)	27.80 (25.60, 30.80)	27.90 (25.50, 30.80)	-0.594	0.552
Current smoking (n, %)	202(44.01)	57(41.01)	0.391	0.532
Kidney stones (n, %)	30(6.54)	8(5.76)	0.109	0.741
Bone erosion (n, %)	55.12(253)	28(20.14)	52.396	<0.001*
Double-track sign (n, %)	47.93(220)	38(27.34)	18.443	<0.001*
Joint effusion (n, %)	264(57.52)	75(53.96)	0.551	0.458
Affected joints (n)	2.00 (1.00, 4.00)	1.00 (0.00, 1.00)	-13.200	<0.001*
TC (mmol/L)	4.68(4.05, 5.38)	4.54(4.03, 5.13)	-1.247	0.212
TG (mmol/L)	1.66(1.14, 2.40)	1.59(1.20, 2.11)	-0.775	0.439
LDL-c (mmol/L)	2.95 ± 0.84	2.86 ± 0.76	-1.197	0.232
HDL-c (mmol/L)	1.00(0.86, 1.16)	1.00(0.88, 1.21)	-0.922	0.356
non-HDL-c (mmol/L)	3.65(3.06, 4.28)	3.50(3.07, 4.07)	-1.394	0.163
FPG (mmol/L)	5.03(4.50, 5.75)	4.96(4.51, 5.76)	-0.067	0.947
eGFR-scr (ml/min/1.73m^2^)	99.70(81.90, 112.20)	103.90(92.30, 117.50)	-3.165	0.002*
SUA (μmol/L)	461.80 ± 117.65	465.95 ± 125.65	0.359	0.720
CysC (mg/L)	1.08(0.95, 1.33)	0.99(0.88, 1.16)	-4.538	<0.001*

BMI, body mass index; TC, total cholesterol; TG, triglyceride; LDL-c, low-density lipoprotein-cholesterol; HDL-c, high-density lipoprotein-cholesterol; FPG, fasting plasma glucose; SUA, serum uric acid. Student’s *t*-tests, Mann-Whitney *U* tests, or χ^2^ tests were used. ^*^P < 0.05 indicates statistical significance.

### 3.2 Stratified analysis of gout characteristics by serum CysC levels

To further investigate the relationship between serum CysC levels and the presence of tophi, we analyzed the clinical characteristics of gout patients stratified by CysC quartiles (**Table 2**). Compared with patients in the lowest quartile (Q1) of CysC (≤0.93), those in the highest quartile (Q4) of CysC (>1.28) were of advanced age, had a longer disease duration, and were more likely to have current smoking. Notably, the prevalence of tophi increased significantly across ascending CysC quartiles (64.78%, 77.33%, 77.70%, and 88.65% for Q1 through Q4, respectively, P<0.001). The proportion of patients with a history of kidney stones, double contour sign, synovial hypertrophy, and bone erosion also rose with higher CysC levels (P< 0.05).

**Table 2:** Clinical characteristics of Cystatin C quartile stratification in male gout patients.

Variables	Q1(≤0.93)	Q2(0.93-1.06)	Q3(1.06-1.28)	Q4(>1.28)	P- value
Age (years)	43.00(36.00, 54.00)	47.50(33.00, 56.00)	56.00(40.00, 65.00)	60.00(47.50, 68.50)	<0.001^*^
Duration of gout (years)	5.00(1.00, 10.00)	5.00(1.50, 12.00)	8.00(3.50, 13.75)	10.00(4.50, 16.00)	<0.001^*^
BMI (kg/m^2^)	28.40(26.30, 31.70)	28.40(25.68, 31.85)	27.75(25.60, 30.68)	26.70(24.60, 29.50)	<0.001^*^
Current smoking (n, %)	59((37.11))	65(43.33)	57(38.51)	78(55.32)	0.007^*^
Tophus (n, %)	103(64.78)	116(77.33)	115(77.70)	125(88.65)	<0.001^*^
Kidney stones (n, %)	2(1.26)	8(5.33)	10(6.76)	18(12.77)	<0.001^*^
Bone erosion (n, %)	57(35.85)	70(46.67)	71(47.97)	83(58.86)	0.001^*^
Double contour sign (n, %)	65(40.88)	66(44.00)	60(40.54)	67(47.52)	0.597
Joint effusion (n, %)	100(62.89)	81(54.00)	86(58.11)	72(51.06)	0.181
Synovial hypertrophy, n (%)	104(65.40)	96(64.00)	68(45.95)	72(51.06)	<0.001^*^
Number of affected joints	1.00(1.00, 2.00)	2.00(1.00, 3.00)	2.00 (1.00, 3.00)	2.00 (1.00, 4.00)	<0.001^*^

BMI, body mass index. CysC: values were categorized into quartiles, with Q1 representing the lowest and Q4 the highest.

Kruskal-Wallis H-test or χ^2^ test was used. ^*^P < 0.05 indicates statistical significance.

### 3.3 Associations of CysC with joint imaging features and metabolic parameters in gout

In GLMs adjusted for age, duration of gout, BMI, and current smoking status, higher serum CysC levels were significantly associated with a greater number of affected joints, presence of tophi, history of bone erosion, and presence of the double contour sign (all P < 0.05) (**Table 3**). Serum Mg levels were positively associated with serum CysC levels and were inversely associated with fasting plasma glucose levels.

**Table 3:** Association of clinical characteristics with cystatin C levels using generalized linear models.

Variables	β (95%CI)	Wald χ^2^	McFadden’s R²	P- value
Number of affected joints	0.012(0.004, 0.021)	7.93	0.464	0.005^*^
Tophus-free (n, %)	-0.083(-0.130, -0.033)	10.79	0.470	0.001^*^
Without bone erosion (n, %)	-0.061(-0.103, -0.019)	8.13	0.463	0.004^*^
Without double contour sign (n, %)	-0.055(-0.096, -0.013)	6.62	0.458	0.010^*^
FPG (mmol/L)	-0.008(-0.024, 0.008)	0.89	0.440	0.346
Mg(mmol/L)	-0.329(-0.595, -0.063)	5.87	0.455	0.015^*^

BMI, body mass index; FPG, fasting plasma glucose; Mg, magnesium.

GLMs specified with an inverse Gaussian distribution and a log link function were used to analyze the data, adjusting for age, duration of gout, BMI, and current smoking status. ^*^P < 0.05 indicates statistical significance.

### 3.4 Serum CysC as an independent risk factor for tophus formation

We used the lowest quartile of serum CysC (Q1) as the reference group in the logistic regression analysis (**Table 4**). In the crude model, the odds ratios (ORs) for the presence of tophi in Q2, Q3, and Q4 were 1.86 (1.12–3.06), 1.90 (1.14–3.14), and 4.25 (2.30–7.85), respectively (all P < 0.05). After adjustment for potential confounders (including age, duration of gout, BMI, SBP, DBP, TC, TG, LDL-c, HDL-c, FFA, Mg, Scr, SUA, FPG, current smoking status, kidney stone, synovial hypertrophy, and double contour sign), the associations remained statistically significant for Q2 and Q4, with adjusted ORs (95% CI) of 1.71 (1.04–3.09) and 3.87 (1.64–9.16), respectively (both P < 0.05). Although the OR for Q3 was 1.65 (95% CI, 0.91–3.00), it did not reach statistical significance (P = 0.098). A significant dose–response relationship was observed across CysC quartiles in both unadjusted and adjusted models (P for trend < 0.05).

**Table 4:** Unadjusted and multivariate adjusted ORs of the quartiles of CysC for participants with tophus.

CysC Quartiles	Unadjusted model		Adjusted model	
	OR (95%CI)	P-value	OR (95%CI)	P-value
Q1(≤0.93)	Reference	–	Reference	–
Q2(0.93-1.06)	1.86(1.12, 3.06)	0.016^*^	1.71(1.04, 3.09)	0.037^*^
Q3(1.06-1.28)	1.90(1.14, 3.14)	0.013^*^	1.65(0.91, 3.00)	0.098
Q4(>1.28)	4.25(2.30, 7.85)	<0.001^*^	3.87(1.64, 9.16)	0.002^*^
*p* for trend	–	<0.001^*^	–	0.003^*^
CysC*Mg (interaction)	3.81(1.75, 8.28)	<0.001^*^	3.00(1.26, 6.96)	0.013^*^

Logistic regression analysis: Adjusted for age, duration of gout, BMI, SBP, DBP, TC, TG, LDL-c, HDL-c, FFA, Mg, Scr, SUA, FPG, current smoking, presence of kidney stones, synovial hypertrophy, and double contour sign. CysC: values were categorized into quartiles, with Q1 representing the lowest and Q4 the highest. The interaction for CysC and Mg: adjusted for age, duration of gout, BMI, TC, TG, LDL-c, SUA, FPG, current smoking status, presence of kidney stones, synovial hypertrophy, and double contour sign. ^*^P < 0.05 indicates statistical significance.

OR (95%CI): odds ratio (95% confidence interval); BMI, body mass index; SBP, systolic blood pressure; DBP, diastolic blood pressure; TC, total cholesterol; TG, triglyceride; LDL-c, low-density lipoprotein-cholesterol; HDL-c, high-density lipoprotein-cholesterol; FFA, free fatty acid; Mg, magnesium; SUA, serum uric acid; Scr, serum creatinine; FPG, fasting plasma glucose; CysC, Cystatin C.

Notably, patients in the highest quartile of serum CysC (>1.28 mg/L) had a 3.87-fold higher odds of tophus formation compared with those in the lowest quartile (≤0.93 mg/L), after adjustment for potential confounders. These findings suggest that elevated CysC levels, as a marker of impaired renal function, may be associated with an increased risk of tophus development. In addition, a significant interaction between CysC levels and serum Mg was observed (p for interaction < 0.001), which remained robust after multivariable adjustment. Stratifying patients by median serum magnesium levels (≤0.87 mmol/L vs. >0.87 mmol/L), elevated CysC remained significantly associated with higher risk in the lower Mg group (OR = 2.65, 95% CI 1.16–7.16), while the association in the higher Mg group was weaker (OR = 2.72, 95% CI 0.93–10.01). These findings suggest that serum Mg status may influence the association between CysC and tophus burden, pointing to a potential modulatory role of Mg in the pathogenesis of tophus formation among patients with gout.

To further investigate the relationship between CysC and tophus formation, we performed subgroup analyses stratified by age at onset, smoking status, diabetes, and dyslipidemia (**Supplementary Figure 1**). The results indicated that none of these factors materially modified the association between CysC and tophus, with all interaction P values exceeding 0.05.

Multivariate ordinal logistic regression analysis identified longer gout duration, presence of bone erosion, presence of the double contour sign, and higher serum CysC levels as independent risk factors for tophus formation (**Table 5**). Compared with patients who had multiple sites of bone erosion, the ORs for tophus were 0.23 (95% CI, 0.13–0.40; P < 0.001) in those without bone erosion and 0.53 (95% CI, 0.30–0.95; P = 0.032) in those with single-site bone erosion. These findings suggest that longer disease duration, structural joint damage, and elevated CysC levels are significantly associated with increased tophus burden in patients with gout.

**Table 5:** Multivariate logistic regression analysis of factors influencing the number of tophi in patients with gout.

Variables	B	SE	Wald	P	OR (95%CI)
Tophus-free	-1.93	1.54	1.57	0.210	0.15(0.01 2.97)
Single tophus	0.23	1.54	0.02	0.882	1.26(0.06,25.63)
Basic clinical characteristics					
Age	0.00	0.01	0.25	0.620	1.00(0.98, 1.01)
Duration of gout	0.09	0.01	47.28	<0.001^*^	1.10(1.07, 1.12)
BMI	0.02	0.02	0.99	0.319	1.02(0.98, 1.06)
Current smoking					
No	-0.22	0.17	1.73	0.189	0.80(0.57, 1.12)
Yes	reference				
Kidney stone					
Absent	-0.26	0.36	0.53	0.467	0.77(0.38, 1.56)
Present	reference				
Biochemical parameters					
TC	0.08	0.13	0.38	0.539	1.08(0.84, 1.39)
TG	-0.05	0.05	0.95	0.329	0.95(0.86, 1.05)
LDL-c	0.19	0.17	1.30	0.255	1.21(0.87, 1.69)
HDL-c	-0.31	0.26	1.43	0.232	0.74(0.45, 1.22)
SUA	0.00	0.00	0.22	0.642	1.00(1.00, 1.00)
Mg	-0.19	1.14	0.03	0.869	0.83(0.09, 7.80)
FFA	0.39	0.51	0.60	0.440	1.48(0.55, 4.03)
Scr	0.00	0.00	0.38	0.54	1.00(0.99, 1.00)
FPG	0.06	0.07	0.82	0.365	1.07(0.93, 1.23)
CysC levels					
Q1	-1.10	0.30	13.35	<0.001^*^	0.33(0.18, 0.60)
Q2	-0.71	0.29	5.94	0.015^*^	0.49(0.28, 0.87)
Q3	-0.77	0.27	8.16	0.004^*^	0.47(0.28, 0.79)
Q4	reference				
Imaging features of joint damage in gout					
Bone erosion					
None	-1.47	0.28	26.77	<0.001^*^	0.23(0.13, 0.40)
Single erosion	-0.63	0.30	4.58	0.032^*^	0.53(0.30, 0.95)
Multiple erosions	reference				
Double contour sign					
Absent	-0.67	0.20	11.20	0.001^*^	0.51(0.35, 0.76)
Present	reference				
Joint effusion					
Absent	-0.13	0.18	0.47	0.492	0.88(0.62, 1.26)
Present	reference				
Synovial hypertrophy					
Absent	0.09	0.20	0.21	0.649	1.10(0.74, 1.62)
Present	reference				

B, Partial Regression Coefficient; SE, Standard Error; OR, Odds Ratio; 95% CI, 95% Confidence Interval; BMI, body mass index; TC, total cholesterol; TG, triglyceride; LDL-c, low-density lipoprotein-cholesterol; HDL-c, high-density lipoprotein-cholesterol; SUA, serum uric acid; Mg, magnesium; FFA, free fatty acid; Scr, serum creatinine; FPG, fasting plasma glucose; CysC, Cystatin C.

Ordinal logistic regression analysis was performed, adjusting for age, duration of gout, BMI, TC, TG, LDL-c, HDL-c, SUA, Mg, FFA, Scr, FPG, current smoking status, the presence of kidney stones, bone erosion, double contour sign, joint effusion, as well as synovial hypertrophy, and CysC levels in the model. ^*^P < 0.05 indicates statistical significance. Variable assignments were as follows, Tophi, no tophus, single tophus, multiple tophi. Bone erosion, none, single erosion, multiple erosions. Joint effusion, absent, present. Synovial hypertrophy, absent, present. Smoking, non-smoker, smoker. Kidney stone, absent, present. CysC, values were categorized into quartiles, with Q1 representing the lowest and Q4 the highest.

Furthermore, serum CysC levels demonstrated a strong dose-dependent association with tophus formation. Compared with the highest quartile (Q4), patients in the lowest three quartiles had significantly lower odds of tophus formation, with adjusted ORs of 0.33 (95% CI, 0.18–0.60; P < 0.001) for Q1 (≤0.93 mg/L), 0.49 (95% CI, 0.28–0.87; P = 0.015) for Q2 (0.93–1.06 mg/L), and 0.47 (95% CI, 0.28–0.79; P = 0.004) for Q3 (1.06–1.28 mg/L). These results highlight a clear trend of increasing tophus risk with rising CysC levels.

Spearman correlation analyses showed modest but statistically significant correlations between serum CysC and tophus burden in the bilateral ankle joints (r = 0.170, p < 0.001) and the bilateral knee joints (r = 0.160, p < 0.001). And when stratified by bilateral foot tophus volume (>1 cm³ vs. ≤1 cm³), patients with larger tophi (>1 cm³) had higher serum CysC levels, with median values of 1.13 mg/L (IQR 0.99–1.41) versus 1.03 mg/L (IQR 0.92–1.26) in those with smaller tophi (p < 0.001). Higher serum CysC levels are associated with greater tophus burden in lower-extremity joints, suggesting that CysC may reflect the severity of crystal deposition in gout.

### 3.5 Predictive value of serum CysC for tophus and bone erosion in gout

**Figure 1:**
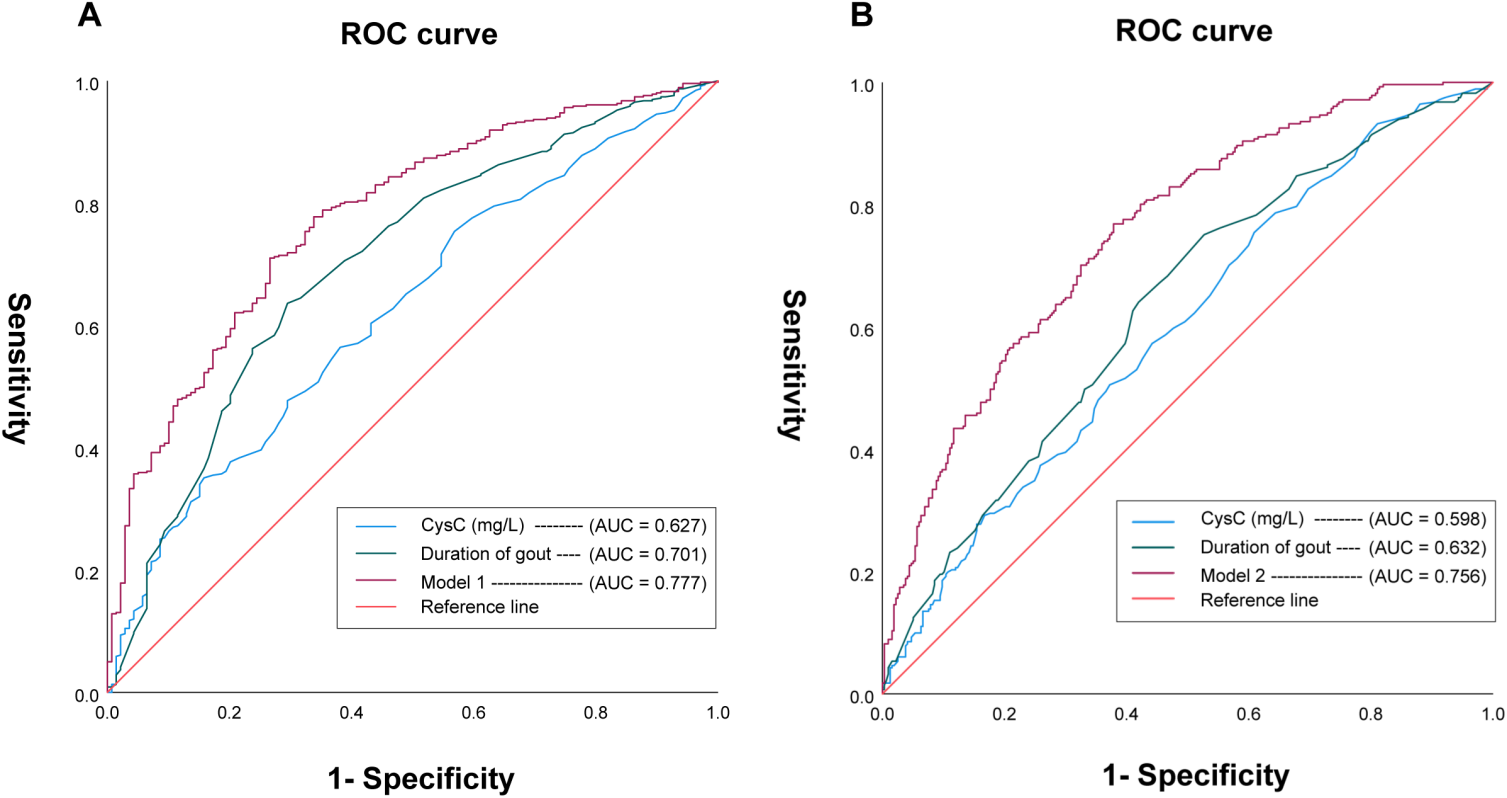
Serum CysC as a predictor of tophus formation **(A)** and bone erosion **(B)** in gout. **(A)** The optimal cutoff of serum CysC for predicting tophus was 1.22 mg/L (blue line), with an AUC of 0.627 (95% CI, 0.58–0.68), sensitivity of 35.1%, and specificity of 84.2% (P < 0.001). Adding gout duration (green line) improved the AUC to 0.701 (95% CI, 0.65–0.75). The model 1 (purple line), which combines CysC, gout duration, BMI, smoking, FPG, Mg, and imaging features (bone erosion, synovial hypertrophy, double-track sign), further improved the AUC to 0.777, with 71.0% sensitivity and 73.4% specificity (P < 0.001). **(B)** For bone erosion, the optimal CysC cutoff was 0.96 mg/L (blue line), yielding an AUC of 0.598 (95% CI, 0.55–0.64), sensitivity of 75.4%, and specificity of 39.1% (P < 0.001). Including gout duration (green line) increased the AUC to 0.632. The model 2 (purple line), incorporating CysC, gout duration, BMI, smoking status, FPG, Mg, and imaging findings (tophus, synovial hypertrophy, and the double-track sign), achieved an AUC of 0.756, with 76.9% sensitivity and 62.1% specificity (P < 0.001).

We used receiver operating characteristic (ROC) curve analysis to evaluate the diagnostic performance of serum CysC for identifying tophus and bone erosion (**Figure 1**). For tophus prediction, the optimal cutoff value of serum CysC was 1.22 mg/L, corresponding to an area under the curve (AUC) of 0.627 (95% CI, 0.58–0.68), with a sensitivity of 35.10% and a specificity of 84.20% (P < 0.001). Among male patients with SUA ≤420 µmol/L, CysC levels >1.22 mg/L were associated with an approximately 2.4-fold higher odds of tophus formation (OR = 2.42, 95% CI 1.11–5.63). And we further incorporated gout duration into the model, which demonstrated predictive value for tophus, yielding an AUC of 0.701 (95% CI, 0.65–0.75). To improve predictive accuracy, a comprehensive multivariable model 1 was constructed by integrating serum CysC levels, gout duration, BMI, current smoking status, FPG, Mg levels, and the presence of bone erosion, synovial hypertrophy, as well as the double-track sign. This model achieved a significantly improved AUC of 0.777 (95% CI, 0.73–0.82), with a sensitivity of 71.00% and a specificity of 73.40% (P <0.001).

In addition, the optimal cutoff value of serum CysC for predicting the presence of bone erosion was 0.96 mg/L. At this threshold, the AUC was 0.598 (95% CI, 0.55–0.64), with a sensitivity of 75.40% and a specificity of 39.10% (P < 0.001). We also further incorporated gout duration into the model, yielding an AUC of 0.632 (95% CI, 0.59–0.68). Moreover, a multivariable predictive model 2 for bone erosion was constructed by combining key variables, including serum CysC, duration of gout, BMI, current smoking status, FPG, Mg, and the presence of tophus, synovial hypertrophy, as well as the double-track sign. This combined model demonstrated improved diagnostic performance, with an AUC of 0.756, sensitivity of 76.90%, and specificity of 62.10% (p < 0.001).

In a sensitivity analysis stratified by eGFR (<90 vs. ≥90 mL/min/1.73 m²), elevated CysC remained significantly associated with an increased risk of tophus formation among patients with preserved renal function (eGFR ≥90; OR = 3.36, 95% CI 1.39–10.07), indicating its independent predictive value.

Calibration curve analysis revealed minimal deviation between the ideal and observed curves for both Model 1 and Model 2 (B = 100 repetitions; mean absolute error = 0.012 and 0.016, respectively), indicating high predictive accuracy (**Supplementary Figures 2A, B**). We further used Decision Curve Analysis (DCA), which demonstrated that both models provided a favorable net benefit, indicating a positive effect in clinical practice (**Supplementary Figures 3A, B**).

Furthermore, to enhance clinical interpretability within the constraints of the study design, we developed a simplified risk scoring tool for tophus prediction based on serum CysC, serum Mg, gout duration, and incorporating the interaction between CysC and Mg. The optimal cutoff value for CysC identified in this study was 1.22 mg/L, with values above this threshold defined as high. Serum Mg was dichotomized at the population median (0.87 mmol/L), with lower values classified as low, while gout duration was retained as a continuous variable. The final scoring algorithm and a web-based interactive calculator (implemented using R Shiny) are provided in **Supplementary File 1**. Clinicians can input CysC, gout duration, and serum Mg values to obtain an individualized risk score, corresponding risk category, and suggested clinical risk interpretation.

## 4 Discussion

Tophi represent deposits of MSU crystals in joints and subcutaneous tissues, leading to recurrent episodes of chronic granulomatous inflammation and serving as a major cause of deformity and disability in patients with gout (19). Although traditionally regarded as a hallmark of advanced gout, tophi may also emerge early in the disease course. Their impact on patients’ overall health status and quality of life is frequently underestimated. Reduced creatinine clearance has been reported to be associated with the early development of subcutaneous tophi in patients with gout (20). In addition to its sensitivity in eGFR, CysC has gained attention for its potential to reflect broader pathophysiological processes beyond renal function. However, its relationship with tophus formation in gout remains unclear (21).

We found that patients with tophi were older, had a longer duration of gout, and exhibited significantly higher serum CysC levels compared with those without tophi, suggesting a potential link between elevated CysC and more advanced or severe disease phenotypes. These patients also showed increased levels of TC, TG, LDL-C, and FPG. Concurrently, creatinine clearance was significantly reduced, indicating that urate crystal deposition in the kidneys may contribute to persistent renal impairment. Although SUA is a key factor in gout pathophysiology, its association with the risk of tophus formation may have been diminished in this study because all participants were hospitalized and received standardized urate-lowering therapy. Notably, our results showed that patients with higher CysC levels had longer disease duration, a greater tophus burden, and more extensive joint involvement, suggesting that elevated CysC may reflect the cumulative effects of inflammation and tissue damage.

Recent studies have suggested that elevated CysC levels may be closely associated with pathological processes, including inflammation, oxidative stress, and endothelial dysfunction. These factors not only contribute to the progression of kidney disease but also play a pivotal role in the development of various cardiovascular and metabolic disorders (22). Cysteine proteases are key enzymes that regulate the turnover and restructuring of the basement membrane and extracellular matrix (23). CysC and cysteine protease dysregulation are closely linked to the development of fibrosis, and growing evidence supports CysC as a promising biomarker for organ fibrosis (24).

In gout, MSU crystals trigger activation of the NLRP3 inflammasome, leading to the release of TNF-α and key proinflammatory interleukins, including IL-1β, IL-6, and IL-18, which mediate acute flares and contribute to chronic inflammation (25, 26). The gene encoding CysC is considered to have prognostic relevance in inflammatory conditions, with inflammatory cytokines modulating its expression. Persistent chronic inflammation may also further promote CysC secretion by stimulating epithelial cells and monocytes (27, 28). CysC is strongly associated with metabolic disorders, serving as a well-established predictor of long-term all-cause and cardiovascular mortality in patients with metabolic syndrome (29). Adjusting CysC could enhance the predictive value of the TyG index for major adverse cardiovascular events in patients with acute coronary syndrome undergoing PCI (30). An analysis of NHANES III demonstrated a significant positive association between higher SUA and CysC levels among adolescents (31). In our study, elevated serum CysC levels were significantly related to an increased risk of tophus formation and greater disease burden in gout patients, independent of other metabolic or inflammatory parameters. Patients in the highest CysC quartile (Q4) had an approximately fourfold higher risk of tophus formation compared with those in the lowest quartile (Q1). In addition, CysC levels were closely correlated with indicators of joint structural damage, including the number of affected joints, bone erosion, double contour sign, and synovial hypertrophy.

Furthermore, we also observed a significant interaction between serum CysC and Mg levels. Maintaining Mg homeostasis is essential for physiological function. In the early phase of bone injury in mice, administration of ionized magnesium could accelerate bone healing by increasing bone volume through the stimulation of osteoblast activity and suppression of osteoclast activity (32). Our previous study suggested that serum Mg serves as an independent protective factor against bone erosion in patients with gout (33). Low Mg levels have been reported to be associated with impaired uric acid excretion and an increased risk of nephrolithiasis, potentially exacerbating gout-related inflammatory responses (34, 35). The synergistic effect of elevated serum CysC and reduced Mg levels may further contribute to tophus formation, warranting deeper investigation in future mechanistic studies. Given the potential role of magnesium in regulating inflammatory responses and bone metabolism, regular supplementation with over-the-counter magnesium preparations may confer clinical benefits in patients with gout. We look forward to further clinical studies evaluating its therapeutic potential in gout-related complications.

In terms of gout burden, both tophi and bone erosion substantially impair patients’ joint function and quality of life. ROC curve analysis showed that serum CysC had greater predictive value for tophus formation than for bone erosion, with an optimal cutoff value of 1.22 mg/L. Gout duration was a strong predictor for both conditions. These findings highlight the importance of comprehensive gout management, including regular monitoring of CysC and SUA levels, adherence to a low-purine diet, weight management, and early intervention—especially in younger patients with early-onset disease. Although serum CysC demonstrated only moderate predictive performance when used alone and may not be sufficient as a diagnostic marker, it can still be considered a complementary biomarker of clinical relevance. Importantly, the integration of CysC with disease duration, metabolic parameters, and imaging features significantly enhanced the prediction of tophus formation (AUC = 0.777, P < 0.001) and bone erosion (AUC = 0.756, P < 0.001). Furthermore, DCA showed that the combined model yielded a greater net clinical benefit compared with strategies of treating all patients or treating none (**Supplementary Figure 3A**).

Several limitations should be noted in this study. First, owing to its cross-sectional design, causal relationships between serum CysC levels and tophus formation cannot be established, and the observed associations should therefore be interpreted as associative rather than causal. In addition, there is currently no established guidance on how CysC levels should be incorporated into clinical decision-making, such as imaging referral (e.g., DECT) or adjustment of urate-lowering therapy, which limits the immediate translation of our findings into routine clinical practice. Moreover, the potential role of CysC in predicting treatment response was not evaluated. As changes in all tophus burden, gout flare frequency, disease progression, or long-term prognosis were not assessed, the utility of CysC in guiding treatment intensity and prognostic stratification remains to be determined. Site-specific DECT-based volumetric assessments are warranted in future studies to evaluate whether the predictive performance of CysC differs by joint location, particularly in weight-bearing joints commonly involved in early gout. Mechanistically, although CysC has been linked to metabolic disturbances, hyperuricemia, and oxidative stress, direct evidence for its involvement in tophus formation and bone erosion in gout is still limited, and the underlying biological pathways remain incompletely understood. Finally, as the present study included only male patients, the generalizability of these findings to female patients with gout is uncertain. In addition, residual confounding from unmeasured factors, including dietary patterns, alcohol consumption, and physical activity, cannot be fully excluded. Future prospective longitudinal studies incorporating diverse populations, comprehensive lifestyle assessments, and longitudinal clinical and imaging outcomes are warranted to validate and extend the present findings.

## 5 Conclusion

In conclusion, this cross-sectional study identified serum CysC as an independent predictor of tophus formation in patients with gout and revealed a potential interaction between CysC and serum Mg levels. Imaging features including tophi and bone erosion, markers of irreversible joint damage, are strongly associated with disease duration. Combined assessment of serum CysC and imaging markers may help identify patients at increased risk of tophus burden, particularly those with early-onset gout.

## Data Availability

The raw data supporting the conclusions of this article will be made available by the authors, without undue reservation.
