# Supplementary material for: Elevated serum cystatin C predicts tophus formation in gout: evidence from a cross-sectional study
Source: Front Endocrinol (Lausanne). 2026 Mar 30;17:1733216. doi: 10.3389/fendo.2026.1733216 (PMC13070796; doi:10.3389/fendo.2026.1733216)
Supplement: Supplementary file 1 [file DataSheet1.docx]

**Supplementary Table 1. Tophus risk scoring system and stratification (recommended clinical cutoffs)**

| Score Range | Risk Level | Patients (n, %) | Tophus Rate (%) | Clinical Recommendation |
| --- | --- | --- | --- | --- |
| 0-10 | Low Risk | 148 (24.7%) | 57.4 | Routine urate-lowering therapy and follow-up; no additional imaging needed at this time. |
| 11-15 | Medium Risk | 68 (11.4%) | 69.1 | Consider joint ultrasound for tophus detection; intensify urate-lowering therapy; evaluate magnesium status. |
| ≥16 | High Risk | 382 (63.9%) | 85.6 | Strongly recommend imaging (ultrasound or dual-energy CT); actively manage tophus and complications; refer to rheumatology/nephrology. |

Risk categories were defined using fixed integer cutoffs (0-10, 11-15, ≥16) based on the distribution of total scores and the observed gradient in tophus prevalence.


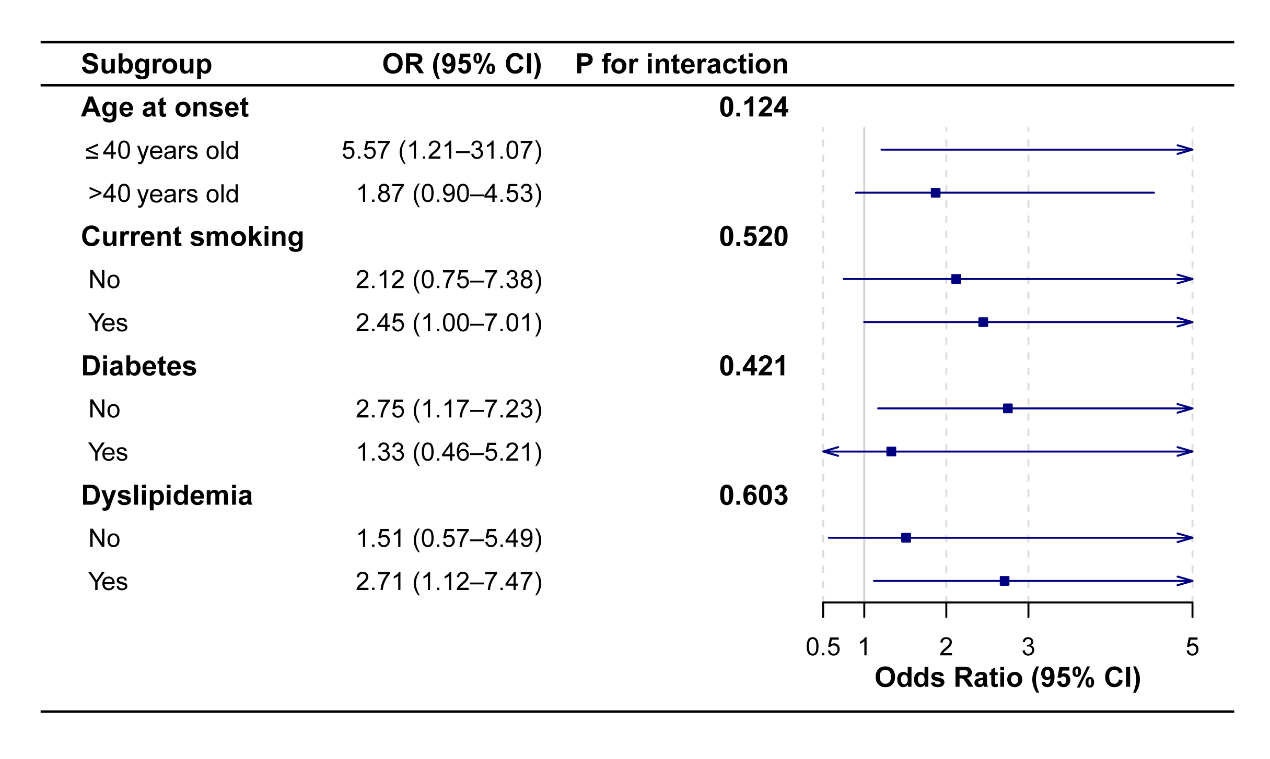


**Supplementary Figure 1. Subgroup analyses showed that age at onset, smoking status, diabetes, and dyslipidemia did not significantly influence the association between CysC and tophus formation (P > 0.05).**


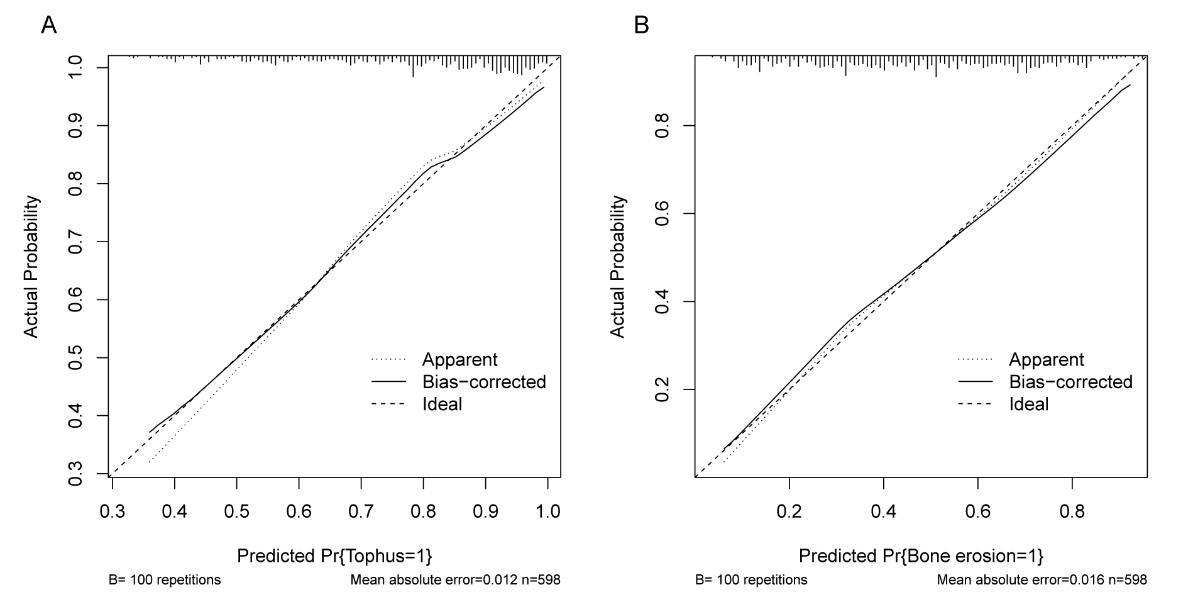


**Supplementary Figure 2. Calibration curves of Model 1 for tophus (A) and Model 2 for bone erosion (B).**


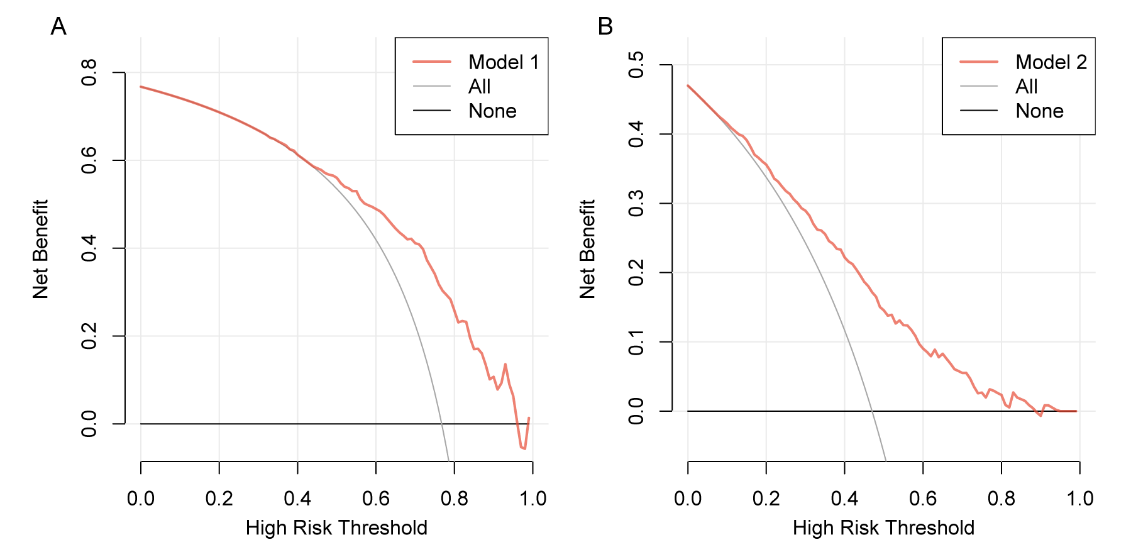


**Supplementary Figure 3. Decision Curve Analysis (DCA) of Model 1 for tophus (A) and Model 2 for bone erosion (B)**


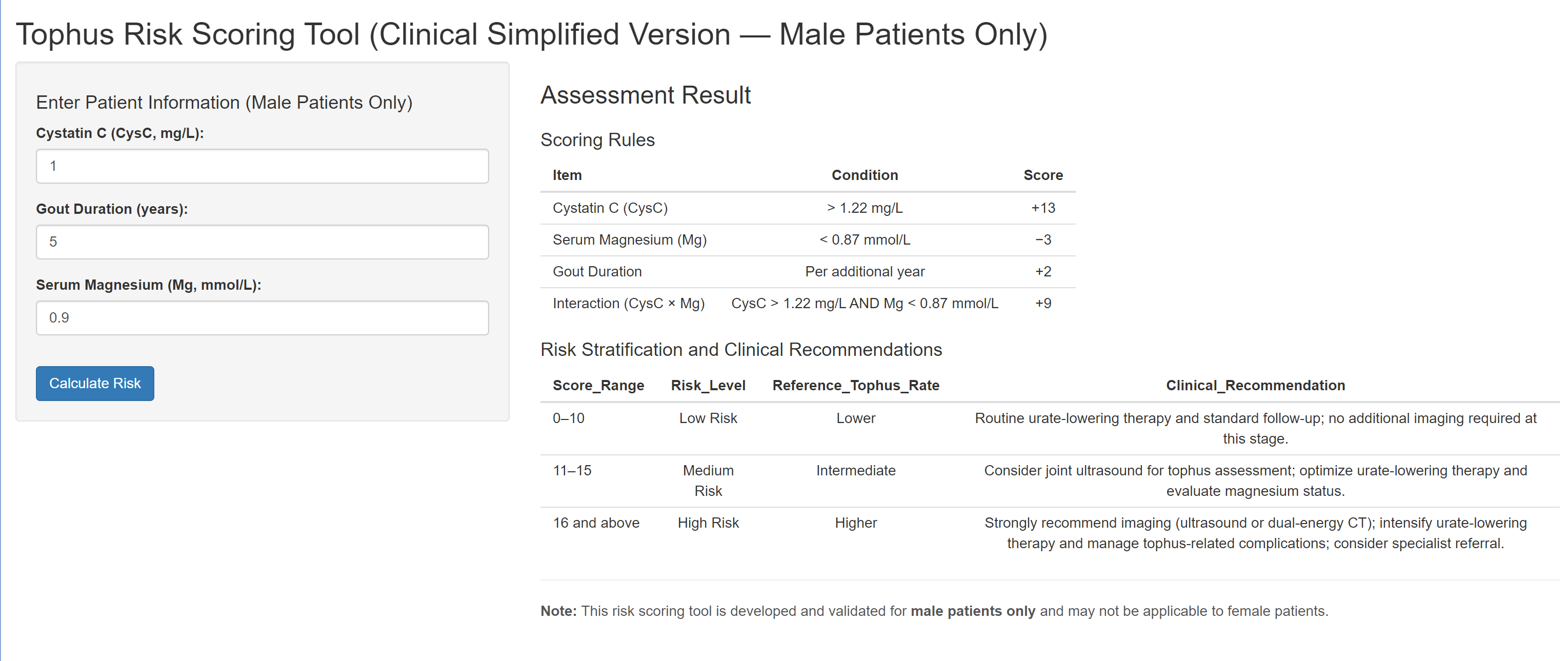


**Supplementary Figure 4. Tophus Risk Scoring Tool interface.**

Users can obtain a risk assessment by entering cystatin C (CysC, mg/L), gout duration (years), and serum magnesium (Mg, mmol/L).

**R language code of Tophus Risk Scoring Tool:**

library(shiny)

# =========================

# Global settings

# =========================

# NOTE:

# This simplified risk scoring tool was developed and validated

# exclusively in male patients and should be applied accordingly.

shift_value <- 5 # Offset to make total score non-negative (fixed)

# =========================

# UI

# =========================

ui <- fluidPage(

titlePanel("Tophus Risk Scoring Tool (Clinical Simplified Version — Male Patients Only)"),

sidebarLayout(

sidebarPanel(

h4("Enter Patient Information (Male Patients Only)"),

numericInput(

"cysc",

"Cystatin C (CysC, mg/L):",

value = 1.00,

min = 0,

step = 0.01

),

numericInput(

"duration",

"Gout Duration (years):",

value = 5,

min = 0,

step = 1

),

numericInput(

"mg",

"Serum Magnesium (Mg, mmol/L):",

value = 0.90,

min = 0,

step = 0.01

),

actionButton(

"calculate",

"Calculate Risk",

class = "btn-primary",

style = "margin-top: 15px;"

)

),

mainPanel(

h3("Assessment Result"),

verbatimTextOutput("result_output"),

h4("Scoring Rules"),

tableOutput("score_table"),

h4("Risk Stratification and Clinical Recommendations"),

tableOutput("risk_table"),

tags$hr(),

tags$p(

tags$strong("Note: "),

"This risk scoring tool is developed and validated for ",

tags$strong("male patients only"),

" and may not be applicable to female patients.",

style = "color: #555555;"

)

)

)

)

# =========================

# Server

# =========================

server <- function(input, output, session) {

# ---- Scoring rules table ----

output$score_table <- renderTable({

data.frame(

Item = c(

"Cystatin C (CysC)",

"Serum Magnesium (Mg)",

"Gout Duration",

"Interaction (CysC × Mg)"

),

Condition = c(

"> 1.22 mg/L",

"< 0.87 mmol/L",

"Per additional year",

"CysC > 1.22 mg/L AND Mg < 0.87 mmol/L"

),

Score = c("+13", "−3", "+2", "+9")

)

}, align = "lcc")

# ---- Risk stratification table ----

output$risk_table <- renderTable({

data.frame(

Score_Range = c("0–10", "11–15", "16 and above"),

Risk_Level = c("Low Risk", "Medium Risk", "High Risk"),

Reference_Tophus_Rate = c("Lower", "Intermediate", "Higher"),

Clinical_Recommendation = c(

"Routine urate-lowering therapy and standard follow-up; no additional imaging required at this stage.",

"Consider joint ultrasound for tophus assessment; optimize urate-lowering therapy and evaluate magnesium status.",

"Strongly recommend imaging (ultrasound or dual-energy CT); intensify urate-lowering therapy and manage tophus-related complications; consider specialist referral."

)

)

}, align = "lccc")

# ---- Calculate button logic ----

observeEvent(input$calculate, {

req(input$cysc, input$duration, input$mg)

cysc <- input$cysc

dur <- input$duration

mg <- input$mg

# ---- Individual score components ----

score_cysc <- ifelse(cysc > 1.22, 13, 0)

score_mg <- ifelse(mg < 0.87, -3, 0)

score_dur <- dur * 2

score_inter <- ifelse(cysc > 1.22 & mg < 0.87, 9, 0)

# ---- Total score ----

total_raw <- score_cysc + score_mg + score_dur + score_inter

total_score <- total_raw + shift_value

# ---- Risk stratification ----

risk_level <- if (total_score <= 10) {

"Low Risk"

} else if (total_score <= 15) {

"Medium Risk"

} else {

"High Risk"

}

suggestion <- switch(

risk_level,

"Low Risk" =

"Routine urate-lowering therapy and standard follow-up; no additional imaging required at this stage.",

"Medium Risk" =

"Consider joint ultrasound for tophus assessment; optimize urate-lowering therapy and evaluate magnesium status.",

"High Risk" =

"Strongly recommend imaging (ultrasound or dual-energy CT); intensify urate-lowering therapy and manage tophus-related complications; consider specialist referral."

)

# ---- Output ----

output$result_output <- renderPrint({

cat("Input Values:\n")

cat(sprintf(" Cystatin C (CysC): %.2f mg/L\n", cysc))

cat(sprintf(" Gout duration: %.1f years\n", dur))

cat(sprintf(" Serum magnesium (Mg): %.2f mmol/L\n\n", mg))

cat("Individual Score Components:\n")

cat(sprintf(" High CysC item: %d\n", score_cysc))

cat(sprintf(" Low Mg item: %d\n", score_mg))

cat(sprintf(" Duration contribution: %.1f\n", score_dur))

cat(sprintf(" Interaction (CysC × Mg): %d\n\n", score_inter))

cat(sprintf("Raw Total Score: %.1f\n", total_raw))

cat(sprintf("Final Total Score (shifted): %.1f\n", total_score))

cat(sprintf("Risk Category: %s\n", risk_level))

cat("Clinical Recommendation:\n", suggestion, "\n")

})

})

}

# Run the application

shinyApp(ui = ui, server = server)
